# A Collaboration Between Game Developers and Rehabilitation Researchers to Develop a Web-Based App for Persons With Physical Disabilities: Case Study

**DOI:** 10.2196/13511

**Published:** 2019-09-06

**Authors:** Alexandra L Terrill, Justin J MacKenzie, Maija Reblin, Jackie Einerson, Jesse Ferraro, Roger Altizer

**Affiliations:** 1 Department of Occupational & Recreational Therapies University of Utah Salt Lake City, UT United States; 2 Department of Physical Medicine & Rehabilitation University of Utah Salt Lake City, UT United States; 3 Department of Health Outcomes & Behavior Moffitt Cancer Center Tampa, FL United States; 4 Entertainment Arts and Engineering University of Utah Salt Lake City, UT United States

**Keywords:** spinal cord injury, software design, interdisciplinary health team, rehabilitation, internet

## Abstract

**Background:**

Individuals with a disability and their partners, who often provide care, are both at risk for depression and lower quality of life. Mobile health (mHealth) interventions are promising to address barriers to mental health care. Rehabilitation researchers and software development researchers must collaborate effectively with each other and with clinical and patient stakeholders to ensure successful mHealth development.

**Objective:**

This study aimed to aid researchers interested in mHealth software development by describing the collaborative process between a team of rehabilitation researchers, software development researchers, and stakeholders. Thus, we provide a framework (conceptual model) for other teams to replicate to build a Web-based mHealth app for individuals with physical disability.

**Methods:**

Rehabilitation researchers, software development researchers, and stakeholders (people with physical disabilities and clinicians) are involved in an iterative software development process. The overall process of developing an mHealth intervention includes initial development meetings and a co-design method called design box, in which the needs and key elements of the app are discussed. On the basis of the objectives outlined, a prototype is developed and goes through scoping iterations with feedback from stakeholders and end users. The prototype is then tested by users to identify technical errors and gather feedback on usability and accessibility.

**Results:**

Illustrating the overall development process, we present a case study based on our experience developing an app (SupportGroove) for couples coping with spinal cord injury. Examples of how we addressed specific challenges are also included. For example, feedback from stakeholders resulted in development of app features for individuals with limited functional ability. Initial designs lacked accessibility design principles made visible by end users. Solutions included large text, single click, and minimal scrolling to facilitate menu navigation for individuals using eye gaze technology. Prototype testing allowed further refinement and demonstrated high usability and engagement with activities in the app. Qualitative feedback indicated high levels of satisfaction, accessibility, and confidence in potential utility. We also present key lessons learned about working in a collaborative interdisciplinary team.

**Conclusions:**

mHealth promises to help overcome barriers to mental health intervention access. However, the development of these interventions can be challenging because of the disparate and often siloed expertise required. By describing the mHealth software development process and illustrating it with a successful case study of rehabilitation researchers, software development researchers, and stakeholders collaborating effectively, our goal is to help other teams avoid challenges we faced and benefit from our lessons learned. Ultimately, good interdisciplinary collaboration will benefit individuals with disabilities and their families.

## Introduction

### Background

Individuals with disability, including spinal cord injury (SCI), traumatic brain injury, and stroke, are at greater risk for mental health issues such as anxiety and depression compared with the general population [1-3]. Yet, findings across the literature consistently document low rates of mental health treatment in these groups. Major barriers exist in accessing effective treatments, including the availability of cost-effective, accessible, and affordable interventions, particularly in more rural areas where transportation barriers may exist [4]. As such, there is a call for improving access, cost, and effectiveness of treatment for individuals with physical disability [5,6].

Mobile health (mHealth) interventions, defined by the World Health Organization as “medical and public health practice[s] supported by mobile phones, patient monitoring devices, personal digital assistants, and other wireless devices,” offer a promising means to overcome many barriers to treatment, including affordability, access, and convenience [7,8]. For example, several mHealth interventions have been developed to provide support for self-management and address physical and psychological needs for individuals with a variety of chronic conditions, including diabetes, depression, and chronic pain [9-14].

Despite the promise and proliferation of mHealth interventions, there is often little attention paid to the design of mHealth tools and apps; the design can be critical to the usability and success of interventions [15]. Research teams interested in pursuing development of mHealth apps may lack critical knowledge of the software development landscape and have limited understanding of how to promote optimal app design [16]. In addition, they may struggle with accessing software developers familiar with the complexities of mHealth, engaging the research population, identifying underlying clinical or research goals, and addressing ethical and legal considerations. Although researchers are well trained in the development of high-quality evidence-based interventions, mHealth apps that fail to address issues of usability and the needs of the target audience will have limited applicability [17].

Although mHealth design can be challenging for the general population, more specialized mHealth solutions may be required to address the unique usability needs of persons with physical limitations, compounding the difficulties. For example, mHealth apps should be compatible with consumer off-the-shelf technologies that support communication limitations, such as eye gaze devices, which is not a standard integration. *Universal design* guidelines and *accessibility-based* approaches have drawn much-needed focus on providing access to technology for individuals with disabilities. However, these frameworks are not sufficient and could even be counterproductive; the inherent variability in needs makes it difficult and unrealistic to develop products for every user. Instead, developers may create products for *average* user accessibility needs, which may further marginalize individuals with disability. Although compliant with accessibility guidelines, resulting products may not be usable by the specific intended audience [18]. Building on universal design approaches, Newell [18] proposed a *user-sensitive inclusive design*, which incorporates a user-centered approach and emphasizes working with target audiences to better understand and design for specific needs. Our framework expands on the user-sensitive inclusive design by emphasizing the relationships between software developers, rehabilitation researchers, and stakeholders as partners to effectively use technology to deliver evidence-based interventions addressing key needs. A user-centered approach and iterative design process are critical to supporting the needs of persons with a variety of disabilities [19], affecting both the efficacy of the intervention and the effective use of the mHealth technology. As such, involving the unique expertise held by rehabilitation researchers, software development researchers, and clinician and patient stakeholders at every stage of the design is critical for success.

Without a shared understanding between siloed areas of expertise, a variety of pitfalls can occur. Teams can experience frustration and conflict, deadlines can be missed, and unexpected costs can be incurred. This is particularly true when collaborators are in different types of institutions, for example, universities and private companies. Ultimately, an ineffective relationship between rehabilitation researchers, software development researchers, and stakeholders can and frequently does lead to the development of mHealth apps that do not meet the needs or standards of one or more of these groups. Poor design choices that fail to meet the requirements of the end user, lack positive user engagement, or do not demonstrate evidence of efficacy will be misused or underused and ultimately fail to meet their original objectives [20]. The proposed solution for these pitfalls presents itself in a collaborative partnership, in which rehabilitation researchers, software development researchers, and stakeholders are aware of each other’s goals and expectations and can communicate more effectively. In this way, an idea can successfully become a tangible product.

### Objectives

The objective of this study was to describe the collaborative process between a team of rehabilitation researchers, software development researchers, and stakeholders with unique areas of expertise. We provide a framework to guide the creation of a Web-based mHealth app intended for individuals with physical disability. We first describe the overall process used for app development and then present a case study to describe our specific experience.

## Methods

### Participants and Team Members

**Figure 1:**
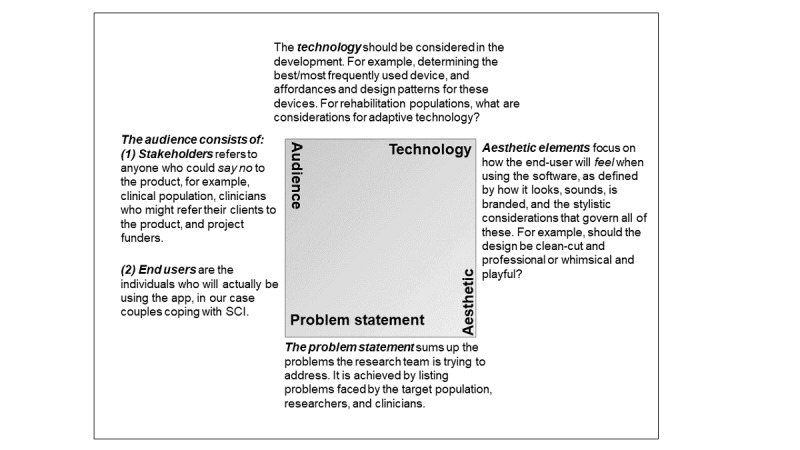
Design box. SCI: spinal cord injury.

As previously noted, the key to the proposed app development approach is a collaborative partnership between 2 research teams and audience, which consists of stakeholders and end users (Figure 1).

Rehabilitation researchers included a team of 3 clinical researchers and a social scientist with expertise in relationships, behavioral interventions, communication, family care partners, positive psychology, intervention development, activity analysis, and adapting technologies to meet functional ability needs. The clinical researchers provided expertise for individuals with physical disabilities, specifically SCI for purposes of this project.

The members of this software development research team worked in a game and app development laboratory located on a health sciences campus. This laboratory offers specialization in inductive and co-design methods with expertise in development of patient and health system facing games and apps. The team included faculty, a project manager, student artists, engineers, and producers.

Individuals with SCI and care partners were included as stakeholders and end users. Stakeholders also included clinicians and therapists: occupational therapists with specific expertise in adaptive technology and SCI, a SCI rehabilitation physiatrist, and an adaptive recreation program coordinator.

### Process

The software development researchers provided information early on about iterative software development, inductive and participatory approaches to design, and the development pipeline and process. These were all very helpful for rehabilitation researchers as the process has significant differences from developing pen-and-paper interventions.

The first major difference involved ideation, understanding the difference between a pitch and a hypothesis. Unlike deductive methods that start with a hypothesis and involve an experiment to support it, design and development precede the hypothesis. In other words, the rehabilitation researchers started the process with an idea, and then, using the design box process (see Figure 1), they worked with the software development researchers to explore the design space and come up with a more developed pitch. This developed pitch solved the same original problem but accounted for the technical affordances of Web-based apps and accessible websites, the end user needs, key stakeholder requirements, and the aesthetics (or content and design related to affect) of the project. This ideation process saved the project’s time and money, as instead of building our initial idea, we used a rigorous process that identified hurdles and holes before spending resources on development.

**Figure 2:**
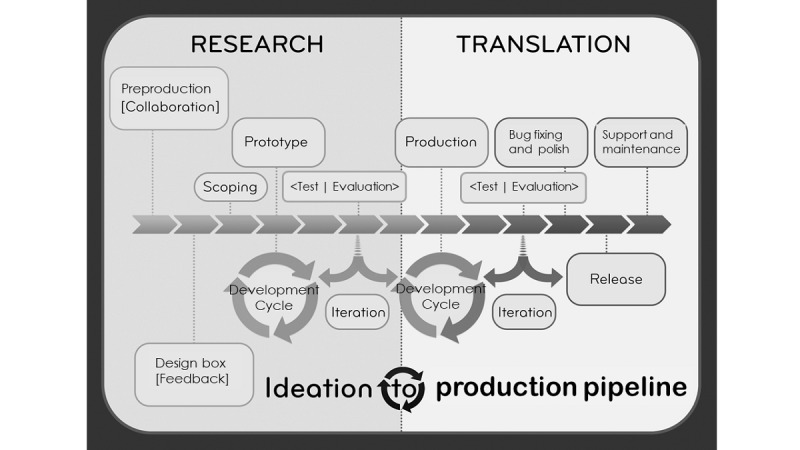
Ideation to production pipeline.

We also created a plan for the development timeline to ensure resources would be available for all stages, including time for stakeholders to contribute design ideas and provide feedback regarding interfaces and processes. We also allocated time for *bug busting* or identifying technical issues. Many rehabilitation researchers fail to realize that making minor changes to the app design often requires additional costs. As many scholars rely on grants, it can be difficult to allocate additional funding to development after the budget has been spent. Initially, the rehabilitation researchers thought that we would go from the initial pitch, to production, to release, not thinking about the other phases. However, we quickly realized that multiple iterations were vital to have a product that was most appropriate for the patient population. In addition, having a software development researcher who was also a faculty member meant that he could relate to the academic roles of the project but still guide the project through development. Figure 2 shows the map we used to pace our time and other resources.

### Collaboration

Important to the collaboration component is creating the culture that unites the rehabilitation and software development teams. As part of the design box, collaborators are encouraged to use “yes, and...” as opposed to “no, but” to foster an open, collaborative environment. This model of positive brainstorming is based on improvisational acting culture as introduced to software development by Randy Pausch and Don Marinelli at Carnegie Mellon [21]. As an inductive and iterative process, the design box aims to elicit ideas from all parties (audience and researchers), allowing for both software that is responsive to user needs and creative. On the basis of the elements identified in the design box, the app developers then propose various solutions.

### Iteration

We iteratively refined the app to meet the end users’ needs while remaining true to the goals of the project.

### Scoping

On the basis of available resources, both in terms of time and funding available, the scope of the project needs to be defined and often redefined throughout the development process.

### Feedback

Stakeholders provide feedback regarding interfaces; contribute to the design ideas; and explain which aspects of mobile interfaces, functions, and tasks they found important.

## Results

### Case Study

**Figure 3:**
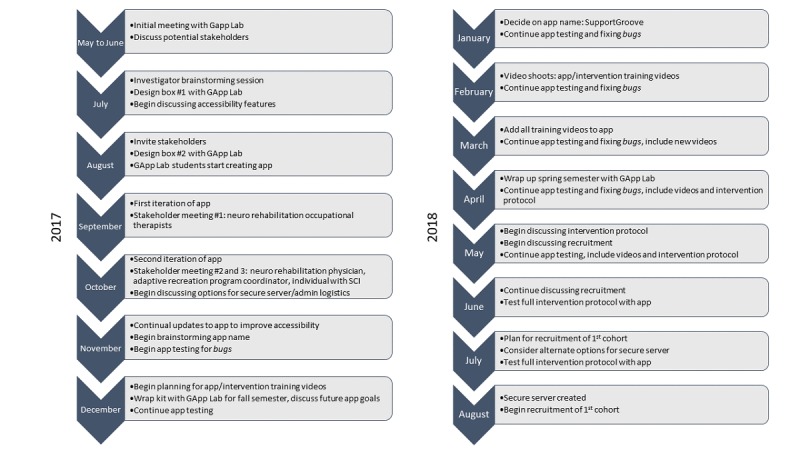
Timeline of project milestones. SCI: spinal cord injury.

The following case study describes the process described above, from initial app ideas to feasibility testing a prototype. Figure 3 shows a timeline of the project milestones. We offer our experience as an example of challenges and solutions.

### Context and Brainstorming

The rehabilitation researchers had previously developed and pilot tested a self-administered pen-and-paper behavioral intervention for couples coping with stroke. The existing intervention protocol, described in more detail elsewhere [22], included a 15-min in-person training session at an outpatient clinic, after which participating couples completed the 8-week intervention on their own, at home, using a paper *activity booklet* that described activities and a paper *tracking calendar* that acted as a log. Activities were based on positive psychology and included expressing gratitude, practicing acts of kindness, focusing on the positive, working toward goals, fostering relationships, savoring, and spirituality. As part of the intervention, participants completed at least four of these 15-min activities per week—2 as a couple and 2 individually—and were asked to log their activity and mood afterward in the pen-and-paper tracking calendar we provided. Participants also received check-in phone calls from a research assistant once a week to remind them to complete activities and send in tracking sheets by mail or email. Although the intervention was generally well received and participants reported finding it beneficial, there were some issues identified by participants and researchers. First, there were accessibility issues; participants were required to travel to the clinic for pre- and postassessments, and they had some difficulties with the pen-and-paper tracking sheet (forgetting to record activities, not having enough space to write, and handwriting was difficult for many participants with hemiparesis). There were also issues with data collection, including missing data, inconsistencies in reporting, and difficulty reading handwritten responses.

Due to these issues and the rurality of our catchment area, moving the intervention to the Web was appealing to the research team. However, with limited app development knowledge, the app initially envisioned by the rehabilitation researchers was limited to what amounted to a direct translation of the existing pen-and-paper intervention, a simple Web page describing activities with video examples. The primary innovation was for the participant to be able to log an activity completed to allow for more reliable tracking. However, during the first meeting with the software development researchers, the rehabilitation researchers were encouraged to *Dream Big* and think about ways to enhance engagement with the app. The rehabilitation researchers were also encouraged to look at existing similar apps for appealing and unappealing features and evaluate user-friendliness.

Some of the initial ideas focused on basic requirements of the app. For example, given the nature of the intervention, it was important for users to have their own space within the app but also the ability to share with a partner. As the intervention focused on doing activities in *real life*, the app was not required to house the actual activities, but rather house the reflections on those activities. Thus, a journaling aspect was desired to allow users to reflect on past activities, and given the target population, the use of voice recordings or pictures was discussed. Other ideas were brainstormed, including gamification of the intervention to enhance engagement, adding an avatar, and getting trophies when activities are completed. On the basis of other apps, important aspects to design also included a simple and clean interface, being able to share/engage in a familiar way, requiring little typing or text, and providing ideas for activities. Although not all ideas were included in the final app, this process helped the rehabilitation researchers to fully consider the options of what this app could be.

### Design Box

Following the initial *brainstorming*, 2 design box meetings were held to identify the problems rehabilitation researchers were trying to address, type of technology to use, who stakeholders and end users were, and what aesthetic elements would look like.

The first design box meeting was held with rehabilitation researchers and the software development faculty and project manager. To facilitate collaboration between the rehabilitation and development teams and establish parameters for the app, the design box meeting started with rehabilitation researchers establishing what needs or problems they were trying to solve. Importantly, this did not include providing potential solutions. For example, there are various unmet needs in the SCI population. The 3 main needs addressed by this team included (1) high rates of depression and lower well-being in individuals with SCI and their partners, (2) limited accessibility of treatments that support mental health/well-being, and (3) little support for partners post-SCI. The rehabilitation researchers were also encouraged to describe what problems they had encountered with the previous pen-and-paper intervention projects, for example, inconsistent or unreliable data collection and missing data. All these *problems* were then distilled into a single *problem statement*: “Current self-reporting solutions that address the well-being of SCI patients and partners are not effective in encouraging personal or dyadic driven activity engagement, nor measuring and recording useful data of a patient’s and partner’s well-being.”

The second design box was held with rehabilitation researchers and all members of the software development research team (faculty, manager, and graduate student team). Once again, rehabilitation researchers provided a list of problems that the intervention app would address, highlighting accessibility, which included remote/rural access, problems identified with pen-and-paper completion, and inclusion of the care partner.

On the basis of design boxes 1 and 2, a *solution statement* was formulated: “The app provides a simple and clear web-based platform where patients and care-partners can log their experiences doing well-being activities, track how they feel, and interact on a daily basis, and identify positive and negative trends during recovery with tools for clinicians to collect and interpret data and progress.” The development of the app was focused on taking actions to address these goals in the solution statement.

### Stakeholders

**Figure 4:**
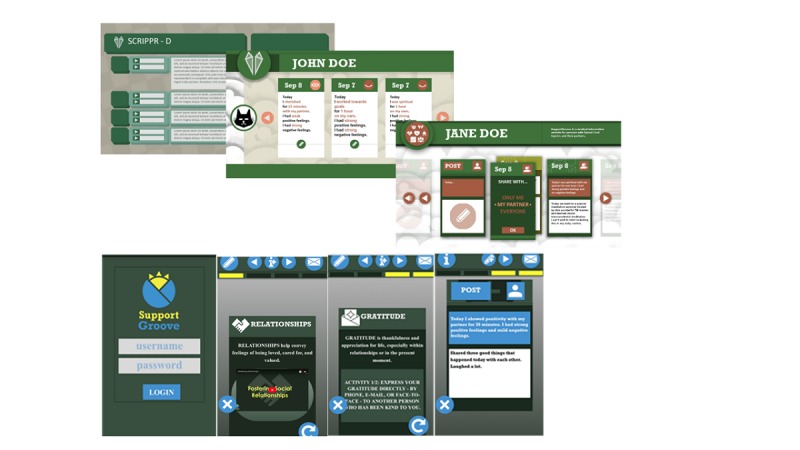
Stakeholders provided feedback on initial designs (above), revealing accessibility issues. Final prototype (below) included minimal scrolling, single click, and large buttons.

In addition to defining our problem statement, another guiding principle was the inclusion of key stakeholders to provide feedback throughout the design process. These stakeholders were identified as content experts (clinicians) and intended end users willing to provide feedback on the app design, accessibility concerns, and usability issues. The rehabilitation researchers and software development researchers met on multiple occasions with stakeholders to discuss early iterations of the project and later to examine and test the app. Inclusion of both the rehabilitation researchers and software development researchers in stakeholder meetings enhanced appreciation of end user accessibility and usability barriers [18]. Feedback and ideas from stakeholders allowed improvement of app iterations (as shown in Figure 4), including ease of use, access, and interface with supportive technologies generally used among persons with SCI.

### Addressing Challenges

In the development of the app, the research team faced key challenges beyond which activities to include in the app. These included data security, remote accessibility, mobile device logistics, and choosing aesthetic elements. Although peripheral to the app activities themselves, these aspects had important implications for the success of the app.

### Security Aspects

Most general apps on the market do not obtain sensitive data; thus, many app developers may not be aware of additional security requirements for mHealth apps. Furthermore, regulatory guidelines and best practices vary across disciplines and location, and understanding what they are and how to apply them can be confusing [23]. mHealth apps developed for research often collect protected personal information, such as names or addresses. Even psychosocial information not considered protected, such as mood ratings, is still sensitive; thus, consent, privacy, and confidentiality are important requirements.

The most important thing teams can do to protect participant data is to make users aware of what data are being obtained and what will be done with it. Many existing commercial mHealth apps can bring in data, such as photos or location, from a variety of mobile device sensors and may also send or sell data to third-party companies, including high-risk data, without the awareness or explicit consent of users [24]. Beyond purposeful sharing of data, teams should also be mindful of making data available only to authorized users. Especially on mobile devices, it is important to have secure log-ins to ensure only the intended user can access the app. This is important for privacy protection and ensuring research teams are collecting valid data from the correct user. In addition, research teams are responsible for protecting data on the back end; this includes storing data on secure servers and encrypted data transfer [24].

Our research team benefited from being housed in a university setting, where regulatory professionals were available to advise us and where a secure infrastructure (eg, encrypted, secure data collection tools, and Health Insurance Portability and Accountability Act of 1996 [HIPAA]-compliant server) was already available. However, understanding which regulations were applicable and ensuring the protections were in place to mitigate risks was still a challenge. Our research team reached out to several offices on campus before we were able to find the right people to help identify and meet our needs. In addition, we had to consider how much protected personal information to include within the app itself. As we were interested in collecting demographic, health, and psychosocial information for research, above and beyond what was critical to the tool, we elected to use Research Electronic Data Capture (REDCap) for our questionnaires [25,26]. REDCap (project-redcap.org) is a data collection and management software used by universities; a major advantage to this was that the security of the server and security of the data were already well managed institutionally. Although our app itself is hosted on a HIPAA-compliant server and the data collected are encrypted, we wanted to ensure that the most sensitive data were even better protected.

### Remote Accessibility

Remote accessibility is a key part of the development of this app. As a core purpose of this app is to promote accessibility for individuals who are unable to meet in person because of disability or location (eg, rural), a focus on remote accessibility is imperative*.* To address this, instructions for how to use the app are emailed, and videos are embedded in the app to provide instructions on how to complete the activities. REDCap is not only used to securely gather data through pre- and postassessments but also to send automated weekly reminder emails. Brief surveys are embedded in the app to collect data on mood every week. We also include a *Contact Us* button in the app. Finally, our pilot study protocol includes in-person or virtual meetings with participants, if needed, to troubleshoot the app.

### Mobile Device Logistics

Given our focus on accessibility, there were other technology specifications that we had to consider for the SCI population. One of the first decisions was to determine the type of device to use as this would drive many of the other decisions. On the basis of demographic statistics on mobile phone use and feedback from stakeholders, we determined that most individuals with an SCI have access to a mobile phone, generally enabled with accessibility options, but do not necessarily have as easy access to a computer. Moreover, most people generally carry a phone with them everywhere they go, increasing ease of activity logging anytime rather than having to wait until they have access to a computer. Although the mobile phone is the preferred device for displaying the app, we decided that a Web-based app would be more appropriate than a downloadable native app to ensure the intervention could be accessed from any connected device.

### Technology

Various accessibility options we initially discussed included eye gaze, switches, large buttons, text to speech, speech to text, mouthstick, control from a power chair (eg, Bluetooth), and drop-down menus. However, including all these options would have exceeded our budget and timeline. Considering input received from stakeholders, we decided to target accessibility for individuals with higher-level spinal cord injuries (eg, injury above the sixth cervical vertebrae, C6), rather than those with lower-level injuries, because those with higher-level injuries generally have more mobility restrictions. Our main design goal was *simpler is better* so that in the future, more accessibility features could be added. For example, we minimized the number of buttons to push when navigating the app and minimized open-ended responses as we found these are often difficult to navigate with accessibility features in other apps.

### Aesthetic Elements

The previously described choices also fed into the choices surrounding the aesthetics of the app. For example, the overall aesthetic *feel* of the app was intended to be polished and clean, which also coincided with the practicality of using adaptive technology and the desire for the app to be a calming experience. Other apps may want to elicit a more *silly* or *energetic* feel. The color palette for the app (colors from nature) was selected based on our aesthetic preference for a more calming experience as well as wanting to make the app useable for those who are colorblind. The nature theme and desire for simplicity also extended to our logo.

Choosing an app name was a somewhat unanticipated challenge. Our goal was to convey the purpose of the app in a concise way. However, it was also important to consider the availability of the domain name and social media handles for branding purposes. Furthermore, we wanted to avoid any potentially insensitive connotations, for example, steering away from “Stepping Up” for an app designed for someone with SCI. The team used a positive brainstorm idea in which it declared a set of values and had team members quickly write words on a whiteboard similar to a free association process. Once the team had exhausted the words or reached theoretical saturation, they attempted to draw connections between them to come up with a name.

Existing branding may also dictate choices in colors and even app names to conform or coordinate with standards or aesthetics of various institutions. For example, if a university or funder logo will be featured prominently within the app, the app aesthetic should not clash with the color scheme. Similarly, some institutions may develop a suite of apps; the names of these apps should also coordinate with each other, yet distinguish themselves. In addition, if there is the intention of letting other universities use it, a process called *white labeling* will allow other institutions to replace it with their branding.

### Play Testing

To make sure the app was as close to a working prototype as possible, we had a number of individuals, including stakeholders, test the app. This included following verbal and/or written instructions, providing detailed feedback of things they liked/did not like, and providing feedback about features that were not working correctly (included sending us screenshots). We asked them to focus on the more mechanical aspects but also asked for general feedback about aesthetic qualities. For the initial app testers, we gave them as little information as possible before testing the app to see how intuitive the app was to use. After making changes as necessary following this feedback, we then gave new testers instructions similar to what we will give participants to test both the app and the instructions (eg, if the instructions were clear enough).

## Discussion

### Building More Effective and Accessible Mobile Health App Interventions

mHealth is a promising way to deliver behavioral health interventions to high-need and high-risk populations, including those with physical disabilities who are otherwise unable to easily access health care resources. However, there are mixed findings concerning the effectiveness of existing mHealth apps. Although some of the problems may be because of ineffective interventions, the implementation of these interventions as apps may also be important to consider. A primary reason why mHealth apps can be poorly implemented is a lack of specialized knowledge, understanding, and communication between rehabilitation researchers, software development researchers, and stakeholders. A collaborative process is key to mHealth; we share our key lessons learned to encourage other research teams in their own work. Our team’s next goal is further refinement of the app based on participant evaluation and eventual broader dissemination.

### Key Lessons Learned

On the basis of our experience, we have provided a summary of lessons learned (see Textbox 1 for a brief overview).

**Brief guide to success: key lessons learned.**

Key lessons learned:

Establish clear guidelines and ground rules for the process:

- Researchers and developers jointly establish clear goals and timelines
- Researchers, developers, and stakeholders establish shared goals
- Make joint decisions about scoping and modifying goals
- Open communication

Understanding stakeholder and end user needs:

- Early and regular engagement is key
- Include those with clinical content expertise and lived experience
- Obtain iterative input
- Understand and use strengths of all members of the research team

Less may be more:

- Clean design and universal design may sometimes not meet specific needs of the end user
- Usability is key

Importance of testing:

- Researchers leverage expertise against design
- Developers test app against common best practices
- Stakeholders and end users test for usability and provide valuable input on specific needs
- Tech-savvy and nontech-savvy playtesters identify bugs and determine how intuitive the design is

Planning ahead:

- Pipeline for publishing software varies at different institutions
- Delays are not uncommon

#### Establishing Guidelines and Ground Rules for the Research and App Development Process

Although our software development researchers are familiar with the research process, we had an introduction meeting where everyone shared and explained their goals. Importantly, partners often may not know what they do not know. As an example, rehabilitation researchers frequently do not know the software development pipeline when developing an app, and software development researchers often do not know the clinical needs. By clearly describing both the research goals and timelines as well as the developer goals and timelines, everyone is better able to understand the mission and what is feasible early on, including where goals differ and overlap. Similarly, the process should also be described to stakeholders. It is important to know the goals of partners and stakeholders involved, and communication should be ongoing to ensure the project continues to move toward shared goals. It was important for everyone’s input to be considered and to make joint decisions about scoping and modifying goals as necessary to meet time and resource demands/limitations. Without knowing why people are asking for things, conflict can arise. In our team, rehabilitation researchers and software development researchers are partners in the development process as opposed to having a developer-client relationship more commonly found in the industry. Being partners allowed for a more collaborative process with effective mutual problem-solving. We recognize that this may not be available to everyone; however, it should be noted that clear expectations for the rehabilitation-developer partnership need to be established early for the project to be successful. By initiating the conversation early, expectations for partnership are established, and the door is opened to continued communication.

#### Understanding Stakeholder and End User Needs and Context

Engaging stakeholders and end users early and regularly from initial design ideas to prototype testing is critical. Inclusion of representatives from all partners (rehabilitation researchers, software development researchers, and various stakeholders) is important to fully appreciate accessibility needs of the end users [18], such as identifying specific app features and hardware that are most preferable, acceptable, and compatible with end user needs. In addition, listening to clinicians and patients is important to get a sense of what fits in clinic workflows or practices as well as daily routines and physical requirements of patients (eg, colorblind and eye gaze). The research team should possess knowledge, skills, and resources to develop and implement the mHealth solution developed with the input provided by the end users in an iterative process. We kept an updated backlog of features and improvements, some of which we were able to address immediately and some of which we needed to resource for future updates.

As a consequence of this understanding, team members learn the other disciplines’ *soft skills*, for example, software development researchers learning about person-first language and rehabilitation researchers learning about the possibilities and pitfalls of software development. However, *hard skills* are kept within one’s own discipline; software development researchers will not be providing psychotherapy, and rehabilitation researchers will not be writing code. This mutual understanding facilitates coherence within the project while supporting unique professional identities and responsibilities. Importantly, there is a synergy in interdisciplinary collaborations that result in better ideas, questions, and solutions than by any one single discipline.

#### Less May Be More

Often teams struggle with fitting more on the page versus simplicity in design to meet usability requirements. Our group often went beyond universal design principles to a user-sensitive inclusive design to include engagement from individuals with varying abilities and needs. Although even those without disability and the need for adaptive technology can appreciate clean design, sometimes there need to be compromises to better meet the needs of specific populations. For example, drop-down menus were a solution for us to keep clutter at a minimum, but we needed to pivot to multiple large buttons to be compatible with eye gaze technology.

#### Importance of Testing

The following 3 groups of testing were included: (1) researchers test the app to leverage their expertise against the design; (2) it is important for the software development team to test themselves or have other developers test it against common best practices; and (3) most importantly, to test with end users on a regular basis, who will not only catch things with a fresh set of eyes but also provide the most valuable feedback of what they will and will not use. All testing data should be interpreted by the team at large to determine whether and in what way an item is actionable. Stakeholders and end users should engage with the app during development to better understand the needs of the target population and receive valuable feedback on design elements. Play testing should be conducted to identify bugs and ideally include 4 types of play testers: those who are or are not tech-savvy and those who are or are not familiar with the specific project. During the testing process, only minimal instructions should be provided to play testers to determine how intuitive the design is. Among play testers, varying abilities and needs should be represented.

#### Planning Ahead

Researchers and developers should familiarize themselves with the pipeline for their health system or school. In some places, it might take longer to establish HIPAA-compliant servers and/or run through security checks. Unlike publishing a paper, the pipeline for publishing software can vary greatly from institution to institution. The team should establish timelines and milestone goals early in the process.

### Conclusions

We have developed a guide—from rehabilitation researchers’ and software development researchers’ perspectives—to serve as a model for other teams who are interested in app-based intervention development. Our model is based on the needs of individuals with physical disabilities; however, it can be adapted to develop apps for other populations with other needs. mHealth solutions are cost-effective, yet there is limited research available that supports successful implementation and sustainability of these types of interventions. End user engagement, clinician buy-in, and funder support are necessary to ensure sustainability. This requires an interdisciplinary approach, which can strengthen and improve accessibility of the end product.

## Abbreviations

HIPAA: Health Insurance Portability and Accountability Act
mHealth: mobile health
PI: principal investigator
REDCap: Research Electronic Data Capture
SCI: spinal cord injury
